# Paraneoplastic Syndrome Case Presented As Nystagmus and Ataxia

**DOI:** 10.7759/cureus.55153

**Published:** 2024-02-28

**Authors:** Khaled M Darwesh

**Affiliations:** 1 Internal Medicine, Providence St. Peter Hospital, Olympia, USA

**Keywords:** uterine cancer, cancer complication, cerebellar degene, paraneoplastic cerebellitis, vertigo, paraneoplastic antibodies, malignancy – endometrial carcinoma – uterine bleeding – serous carcinoma, cerebellar-ataxia, paraneoplastic neurological syndromes, downbeat nystagmus

## Abstract

The incidence of paraneoplastic syndrome (PNS) is on the rise, attributed to the growing detection of antibody modalities in both the serum and cerebrospinal fluid (CSF). PNS can occur as different neurological symptoms. The revised guidelines streamline the diagnostic approach but identifying PNS still requires the detection of neurological manifestations concurrent with cancer, along with the presence of specific PNS autoantibodies.

## Introduction

Nystagmus in general is a rhythmic movement of the eyes. Usually indicates nervous system abnormality in the vestibular systems or the brain stem. Furthermore, according to the type of nystagmus, sometimes it occurs as congenital nystagmus, idiopathic or benign paroxysmal positional vertigo due to otolith in the vestibular systems. On some occasions, it indicates serious findings on another disease such as malignancy, multiple sclerosis (MS), brainstem infarction, or Chiari malformation. In our case, was a devastating finding due to metastatic serous carcinoma of the uterus. With cancer there, too many neurological complications can occur. One of the most important indirect effects of cancer is paraneoplastic syndrome (PNS). The pathogenesis of PNS is not fully understood but the most convincing mechanism is that immune-mediated autoantibodies attack the nervous system in the presence of cancer. Therefore, the earliest diagnosis of PNS is important for early diagnosis of cancer.

## Case presentation

A 69-year-old female patient with osteoarthritis, hypertension, and a recent diagnosis of urinary tract infection presented to the emergency department due to being unable to walk because of significant dizziness associated with nausea. Otherwise, the patient was not aware of any other medical problems. The patients did not report any weight loss, vaginal bleeding, losing appetite, shortness of breath, or any other symptoms. These symptoms had an acute onset four days before presenting to the emergency department. The patient was not able to stand due to significant imbalance; therefore, she decided to come to the emergency department. Upon arrival at the emergency department, the patient was found to have intact vital signs, and intact lab work including hemoglobin level, kidney function, liver function, and electrolytes (Table 1).

**Table 1:** Initial blood work, complete blood count, and complete metabolic panel

Component	Result	Normal range
White blood cell	7.80	4.00 - 12.00 K/µL
Hemoglobin	10.2	12.0 - 16.0 g/dL
Hematocrit	31.6%	37 - 47%
Platelets	327	150 - 450 K/µL
Sodium	136	135 - 145 mmol/L
Potassium	4.0	3.6 - 5.3 mmol/L
Chloride	102	98 - 109 mmol/L
CO2	27	21 - 28 mmol/L
Creatinine	0.6	0.6 - 1.2 mg/dL
Urea	11	8 - 24 mg/dL
Bilirubin, total	0.5	0.2 - 1.4 mg/dL
AST (Aspartate transferase)	17	5 - 40 U/L
ALT (alanine transaminase)	8	0 - 60 U/L
Alkaline Phosphatase	90	28 - 126 U/L
Calcium	10	8.5 - 10.5 mg/dL
Glucose	113	65 - 120 mg/dL

**Figure 1:**
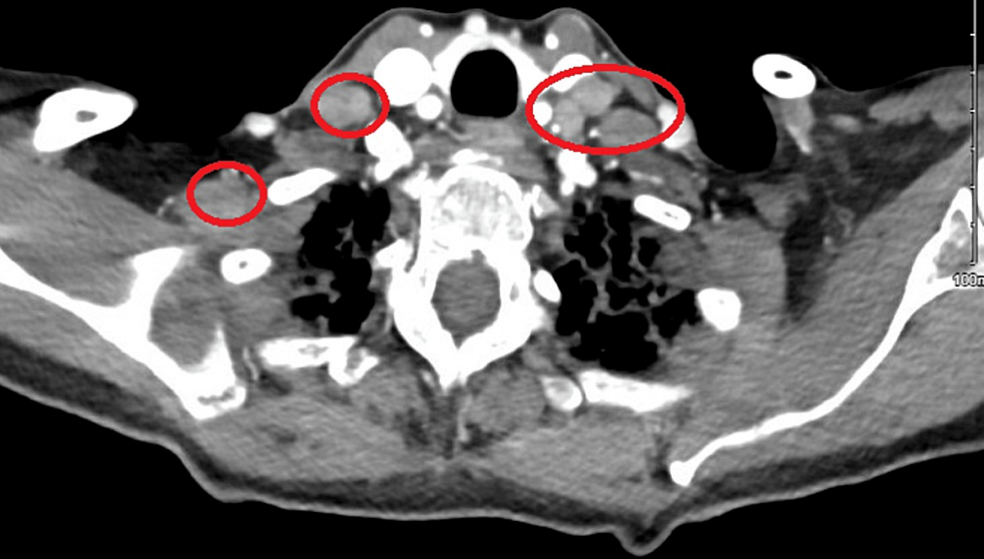
CT neck showed multiple pathologically enlarged and/or pathologic appearing lymph nodes. Supraclavicular lymph node also identified.

The head CT scan performed on the initial presentation is without any evidence of acute intracranial hemorrhage, transcortical infarction or mass. The acute viral panel also is negative for acute findings. Because the head CT scan usually does not rule out acute infarction, especially with a smaller size. Therefore, a brain MRI was ordered which came back with no evidence of acute intracranial infarction, hemorrhage, or mass effect. Also, the brain MRI showed some hyperintensities visualized in the left internal jugular vein which was not specific. Hence, a vascular duplex of the carotid arteries was performed which showed a thyroid nodule of more than 1 cm. Vascular Dopplers of the venous system of the left upper extremity revealed acute superficial thrombophlebitis in the left cephalic vein. Because of these findings in the vascular Doppler, a CT scan of the soft tissue of the neck with contrast (Figure 1) revealed multiple pathologically enlarged lymph nodes in the supraclavicular lymph node enlargement.

**Figure 2:**
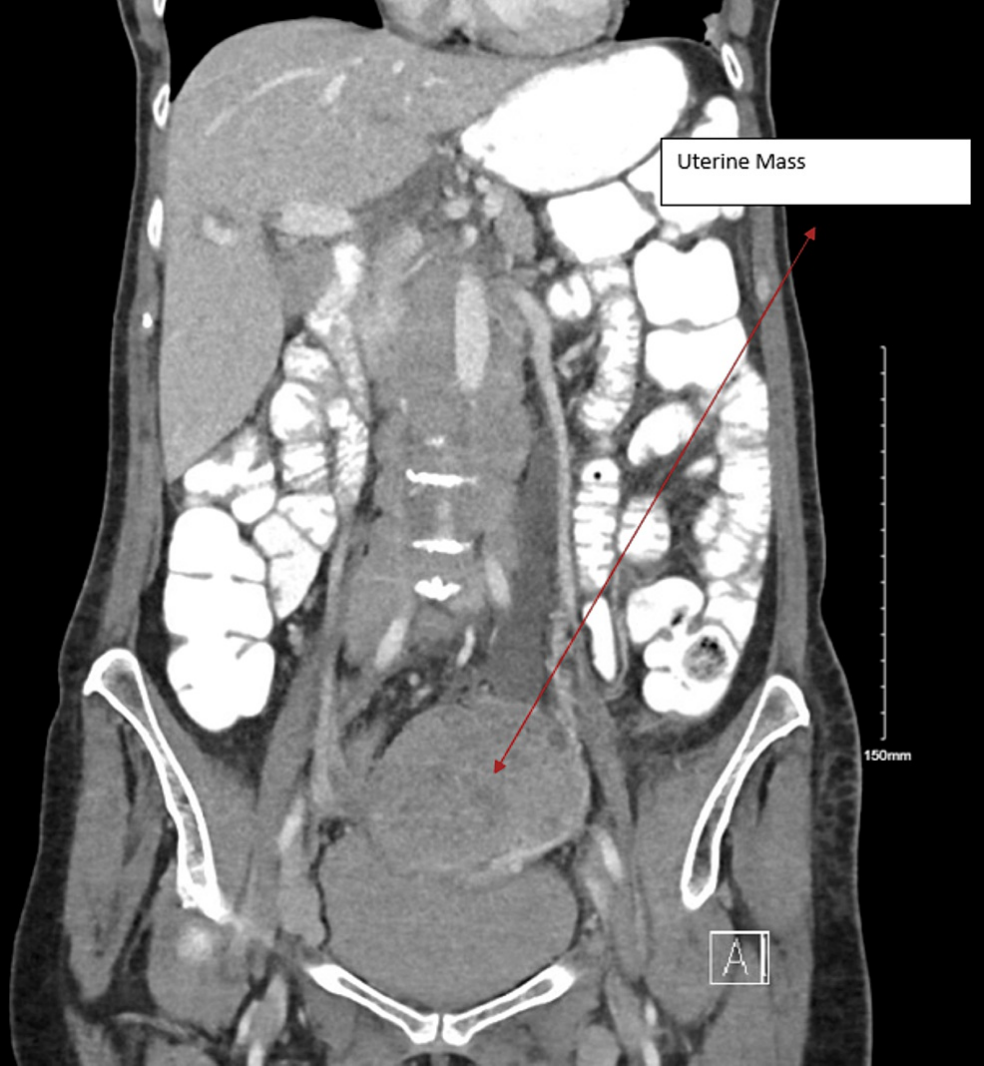
CT abdomen and pelvis

**Figure 3:**
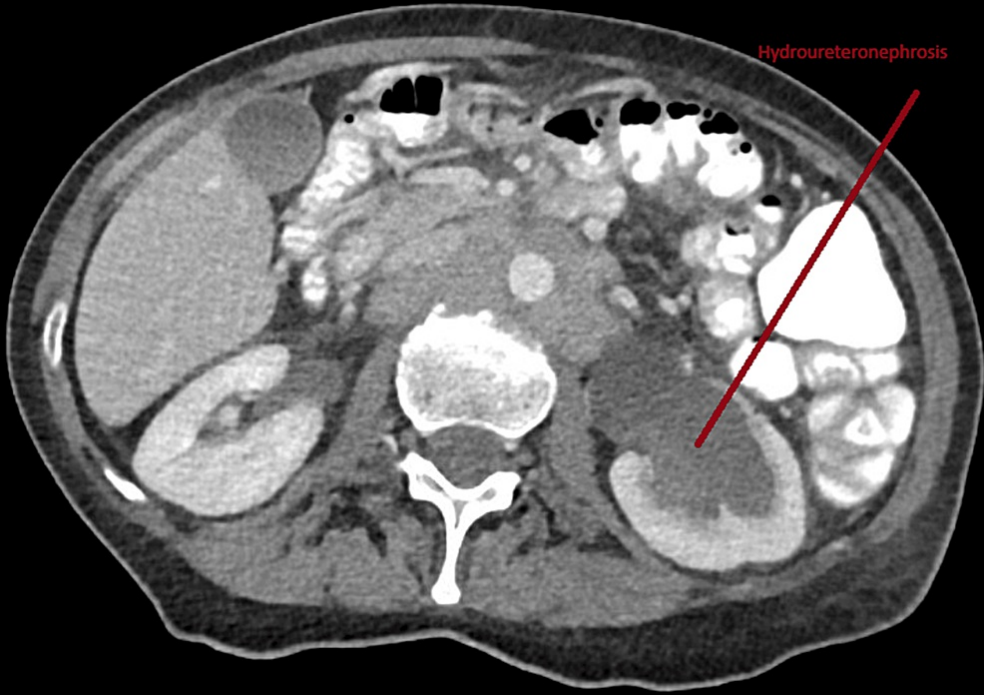
CT abdomen The uterus is enlarged and heterogeneous in appearance with a much larger round heterogeneous mass lesion in the superior aspect of the uterus, 7.1 x 6.0 x 6.8 cm. Extensive periaortic/retroperitoneal bulky lymphadenopathy, encasing the infrarenal abdominal aorta and extending to the bilateral iliac chains. Severe LEFT hydronephrosis and LEFT hydroureter secondary to extrinsic compression on the distal LEFT ureter secondary to the pelvic mass.

The clinical decision was made to do a CT scan of the chest, abdomen, and pelvis with and without contrast. Unfortunately, the result came back with diffuse lymphadenopathies with extensive periaortic, retroperitoneal bulky lymphadenopathy, and bilateral inguinal lymphadenopathies also noted. Additionally, there is a 7.1 x 6.0 x 6.8 tumor in the superior aspect of the uterus. Sadly, this tumor causes pressure on the left kidney and ureter, resulting in left hydrouretronephrosis (Figures 2, 3).

The patient underwent inguinal lymph node biopsy and nephrostomy tube placement to relieve the pressure from her kidney. Neurology consulted and they suggested probably PNS. Further blood testing revealed elevated Ca125 is 4633, and CEA was not significantly elevated. Biopsy from the left inguinal lymph node was consistent with metastatic carcinoma consistent with high-grade serous carcinoma. Due to persistent symptoms of nystagmus and ataxia, a Brain MRI repeated at this time with and without contrast which revealed new subcortical white matter focal foci of T2 prolongation without enhancement along the parietal lobe finding could be due to posterior reversible encephalopathy syndrome; however, it was less likely. The patient was evaluated by gynecology and hemoncology. The gynecologist suggested no surgical intervention given the diffuse metastasis of the malignancy and oncology suggested chemotherapy. The paraneoplastic antibodies panel was sent to the Mayo Clinic, and it took about two weeks for the results to come back. Therefore, the patient started immediately on management with IVIG for five days. After IVIG no significant improvement was seen. The patient continues to work with physical therapy and Occupational Therapy. Unfortunately, she continues to have down-beating nystagmus and ataxia without any improvement. The Neurology suggested treatment is to treat the main etiology, which is malignancy. However, the patient chose to be only in comfort care and discharged home with hospice. Paraneoplastic antibodies came back reactive for Purkinje cell antibodies type I (Anti Yo) came reactive, which is consistent with PNS (Table 2).

**Table 2:** PNS antibodies evaluation

PNS antibodies type	Result (normal is negative)
Anti-Neuronal Nuclear antibodies, Type 1	Negative
Anti-Neuronal Nuclear Antibodies Type 2	Negative
Anti-Neuronal Nuclear Antibodies Type 3	Negative
Ant-Glial Nuclear Ab, Type 1	Negative
Purkinje Cell Nuclear antibodies, Type 1	Reactive
Purkinje Cell cytoplasmic antibodies, Type 2	Negative
DNER: delta/notch-like epidermal growth factor-related receptor	Negative
Amphiphysin Antibodies	Negative
collapsin-responsive mediator protein 5 immunoglobulin	Negative
voltage-gated calcium channel	Negative

## Discussion

No clear mechanism is known for downbeat nystagmus, but it is a type of central vestibular nystagmus resulting from an imbalance in the central vertical vestibulocochlear pathway resulting from damage to one of the two sites in the nervous system, either damage to the dorsal medulla or cerebellar flocculus or its projections, these might be caused by injury to Purkinje cells in the cerebellum which resulted in distribution of ocular movement [1-3]. Etiologies of downbeat nystagmus vary, 25% to 40% of cases approximately are idiopathic. A clinical review study of 62 patients with downbeat nystagmus found the most common cause of downbeat nystagmus where cerebellar ectopia 25% and cerebellar degeneration 25% with another 10% having a variety of conditions. About 40% remain undiagnosed and considered idiopathic [3,4]. In our case, we think the downbeat nystagmus and ataxia are associated with the rapidly progressive cerebellar syndrome as a phenomenon of PNS caused by high-grade serous carcinoma of the uterus. The epidemiology of PNS is not fully clear but it is increasing with the discovery of more diagnostic antibodies. Approximately 3% of small cell cancer might develop Lambert Eaton Syndrome and 15 % of Myasthenia graves incidence in thymoma. In other solid tumors, the incidence is less than 1% [5,6]. The pathogenesis of PNS is not fully understood but the most convincing mechanism is that immune-mediated autoantibodies attack the nervous system in the presence of cancer [1,4]. The theory behind this thought is that cancer cells are different from normal cells by processing self-antigen, for unknown reasons triggering the immune system for autoimmune antibodies reaction. There are two types of neuronal antibodies identified antibodies against intracellular neuronal antigens and antibodies against the neuronal cell surface or synaptic antigens [7]. Antibodies against neuronal cell antigens tend not to respond to treatment. Meanwhile knowing the types of antibodies also will help us with prognosis and management response those patients with PNS. Understanding the criteria for PNS diagnosis is important for early diagnosis of cancer. In too many cases PNS with cerebellar involvement is associated with gynecological malignancy, fallopian tube cancer, and breast and ovarian cancer [8]. Newly updated guidelines added essential help to diagnose PNS. To facilitate understanding PNS diagnosis criteria we need to know antibodies associated with types of malignancy and which neurological phenomenon syndrome can happen (Tables 3-5). Most PNS MRIs are normal especially in the early stages of PNS [9]. The most helpful testing to diagnose PNS is antibodies in serum and cerebrospinal fluid (CSF). We believe our case was the diagnosis most likely rapidly progressive cerebellar syndrome by finding isolated cerebellar symptoms which are ataxia and nystagmus and by reactive serum anti-Yo (also known as PCA-1, Purkinje cell antibody [10]. The diagnostic criteria for PNS were initiated in 2004, defined by two levels of diagnosis as definite and possible [11]. The updated diagnostic criteria for PNS were upgraded in 2021 to three levels of diagnosis definite, probable, and possible. To have a better understanding of the criteria first we need to know the neurologic phenomenon/ syndromes which most likely associated with malignancy. At the same time, these neurological phenomena/syndromes are classified as high risk and intermediate risk in addition to associated paraneoplastic antibodies. High-risk syndromes include Encephalomyelitis, Limbic encephalitis, rapidly progressive cerebellar syndrome, Opsoclonus-myoclonus-ataxia syndrome, Sensory neuronopathy, Gastrointestinal pseudo-obstruction (enteric neuropathy), Lambert-Eaton myasthenic syndrome (LEMS). Intermediates risk. intermediate-risk syndromes include Encephalitis other than well-defined limbic encephalitis, Anti-N-methyl-D-aspartate (anti-NMDA) receptor, encephalitis, Brainstem encephalitis, Morvan syndrome, Isolated myelopathy, Stiff-person syndrome, and polyradiculoneuropathies [7].

**Table 3:** High-risk antibodies, mediated mechanism, neurologic phenomenon, and cancer association ANNA: antineuronal nuclear antibody; SCLC: small cell lung cancer; NSCLC: non-small cell lung cancer; CRMP5: collapsin-responsive mediator protein 5; MG: myasthenia gravis; SOX1: SRY-box transcription factor 1; LEMS: Lambert-Eaton myasthenic syndrome; PCA: Purkinje cell antibody; MAP1B: microtubule-associated protein 1B; DNER: delta/notch-like epidermal growth factor-related receptor; KLHL11: Kelch-like protein 11 Reference [7]

Antibodies	PNS phenomenon	Cancer associated
Hu (ANNA-1) T cell mediated	Sensory neuronopathy, chronic gastrointestinal pseudo-obstruction, encephalomyelitis, limbic encephalitis	SCLC >> NSCLC, other neuroendocrine tumors, neuroblastoma
Yo (PCA-1) T cell-mediated	Rapidly progressive cerebellar syndrome	Ovarian cancer, breast cancer and uterine cancer
Ma2 and/or Ma ) T cell-mediated	Limbic encephalitis, diencephalitis, brainstem encephalitis	Testicular cancer, NSCLC
SOX1 Uncertain if T cell mediated or surface antibodies	with and without rapidly progressive cerebellar syndrome	SCLC
CV2/CRMP5T ) T cell-mediated	Encephalomyelitis, sensory neuronopathy	SCLC, thymoma
Amphiphysin Uncertain; possibly antibody-mediated	Polyradiculopathy, sensory neuronopathy, encephalomyelitis, stiff-person syndrome	SCLC and Breast cancer
Ri (ANNA-2) T cell-mediated	Brainstem/cerebellar syndrome, opsoclonus-myoclonus-ataxia syndrome	NSCLC, SCLC and breast cancer
KLHL11 T cell-mediated	Brainstem/cerebellar syndrome	testicular cancer
Tr (DNER) Uncertain if T cell mediated or surface antibodies	Rapidly progressive cerebellar syndrome	Hodgkin Lymphoma
PCA-2 (MAP1B) T cell-mediated	Sensorimotor neuropathy, rapidly progressive cerebellar syndrome, encephalomyelitis	SCLC, NSCLC, breast cancer

**Table 4:** Intermediate-risk-mediated antibodies mechanism, neurologic phenomenon, and cancer association AMPAR: α-amino-3-hydroxy-5-methyl-4-isoxazolepropionic acid receptor; GABABR: gamma-aminobutyric acid-B receptor; KCTD16: potassium channel tetramerization domain containing; mGluR5: metabotropic glutamate receptor 5; P/Q VGCC: P/Q type voltage-gated calcium channel; NMDAR: N-methyl-D-aspartate receptor; Caspr2: contactin-associated protein-like 2. Reference [7]

Antibodies and mechanism	PNS Phenomenon	Cancer associated
AMPAR Antibody-mediated	Limbic encephalitis	>SCLC, malignant thymoma
mGluR5 Antibody-mediated	Encephalitis	~Hodgkin lymphoma
Caspr2 Antibody-mediated	Antibody-mediated Morvan syndrome, limbic encephalitis	Malignant thymoma
NMDAR Antibody-mediated	Anti-NMDAR encephalitis	Ovarian or extraovarian teratomas
NMDARA Antibody-mediated	Anti-NMDAR encephalitis	Ovarian or extraovarian teratomas
P/Q VGCC Antibody-mediated	LEMS, rapidly progressive cerebellar syndrome	SCLC
GABA_B_R Antibody-mediated	Limbic encephalitis	SCLC

**Table 5:** Low-risk antibodies mGluR1: metabotropic glutamate receptor 1; GABAAR: gamma-aminobutyric acid-A receptor; GFAP: glial fibrillary acidic protein; GAD: glutamic acid decarboxylase; LGI1: leucine-rich glioma inactivated protein 1; DPPX: dipeptidyl-peptidase-like protein; CNS: central nervous system; PERM: progressive encephalomyelitis with rigidity and myoclonus; GlyR: glycine receptor; AQP4: aquaporin 4; MOG: myelin oligodendrocyte glycoprotein; AK5: adenylate kinase 5; GluK2: glutamate kainate receptor subunit 2. Reference [7]

Antibodies / mechanism	PNS phenomenon	Cancer association
GAD65 Uncertain	Limbic encephalitis, stiff-person syndrome, cerebellar ataxia	SCLC, other neuroendocrine tumors, malignant thymoma
GluK2 Antibody-mediated	Encephalitis with prominent cerebellar involvement	Hodgkin lymphoma
GABA_A_R Antibody-mediated	Encephalitis	Malignant thymoma
LGI1 Antibody-mediated	Limbic encephalitis	Malignant thymoma, neuroendocrine tumors
DPPX Antibody-mediated	Encephalitis with CNS hyperexcitability, PERM	Hematologic malignancy
AK5 T cell-mediated	Limbic encephalitis	Unknow type of cancer
GlyR Antibody-mediated	Limbic encephalitis, PERM	Malignant thymoma, Hodgkin lymphoma
MOG Uncertain	myelin oligodendrocyte glycoprotein;	Mostly ovarian teratomas
mGluR1	Cerebellar ataxia	Hematologic malignancy
AQP4	Neuromyelitis optica spectrum disorder	Adenocarcinoma

In Tables 3-5, we outlined associated antibodies with cancer phenomenon and the most common associated cancer. In terms of diagnosis of PNS. PNS is one of too many neurological complications that can occur as an indirect effect of cancer. Diagnosing PNS should rule out another possibility that the cases might cause symptoms such as stroke, brain metastasis, and infectious etiology. As reported in our case the history patient had a sudden onset of nystagmus and ataxia, she is not on any new medications that might contribute to her symptoms such as lithium [12]. The scoring (PNS- Care Score) criteria for PNS is divided into three levels definite, probable, and possible (Table 6).

**Table 6:** PNS-care score Points result explanation: Definite > or equal 8, Probable 6-7, Possible 4-5, Non-PNS Less than or equal 3. Reference [7]

Clinical Level	Points
High risk phenomenon	3
Intermediate risk phenomenon	2
Defined phenomenon epidemiologically not associated with cancer	0
High risk antibodies	3
Intermediate risk antibodies	2
Low risk antibodies	0
Cancer found	4
Cancer not found or not consistent and follow up < 2 years	1
Cancer not found or not consistent and follow up > 2 years	0

Our case scored a definite diagnosis of PNS due to the presence of serous carcinoma of the uterus, ataxia, and nystagmus which indicated rapidly progressive cerebellar syndrome and the presence of reactive Anti Yo is a high-risk antibody [7]. Treatment of PNS is often ineffective, especially in the late presentation of cancer. However, the goal is to treat the cancer and immune-mediated modalities to manage the PNS; with corticosteroids, IVIG, and immune suppressants such as cyclophosphamide and rituximab. PNS antibodies are great guidance to management response and prognosis. Usually, the cases unresponsive to treatment in cases present with PNS antibodies against intracellular antigens in which the mechanism by cytotoxic T cells (Tables 3-5). On the other hand, the treatment is satisfactory in cases with antibodies mediated. One trial with immune modulators with plasma exchange and cyclophosphamide or plasma exchange plus conventional cancer chemotherapy showed a positive response in disability scores. Therefore, this study suggested, even when there is no evidence of malignancy, early consideration of aggressive immunosuppression in patients with PNS symptoms [13].

## Conclusions

PNS is a rare condition induced by the immune reaction to cancer. The first paraneoplastic anti-neuronal antibodies were reported by Wilkinson and Zeromski in 1965. Symptoms vary from peripheral neuropathy, altered mental status, encephalitis nystagmus, or ataxia as occurred in our case. The new guidelines to diagnose PNS indicate a scoring system that presents malignancy, neurological syndromes, and antibodies. Pathogenesis of PNSs occurs by antibodies against either intracellular neural antigens or surface antigens. Usually, the prognosis is worse in the presence of intracellular antibodies. In general, early diagnosis of PNS guides us for management which is cancer management and immunosuppression with steroids, IVIG, cyclosporine, and rituximab in some cases.
